# Association between health literacy and reproductive health knowledge and behavior: A cross-sectional survey among women of reproductive age in Vienna

**DOI:** 10.1007/s00508-025-02672-x

**Published:** 2026-01-03

**Authors:** Pia Rottjakob, Judit Simon, Agata Łaszewska

**Affiliations:** Department of Health Economics, Center for Public Health, Medical University of Vienna, Kinderspitalgasse 15/1, 1090 Vienna, Austria

**Keywords:** Reproductive health, Health education, Women’s health, Reproductive health literacy, Public health

## Abstract

**Objective:**

Despite Austria’s highly ranked healthcare system, health literacy (HL) is lower than in other European countries, with Vienna falling below the national average in life expectancy and healthy life years. Following the 2016 WHO Strategy for Women’s Health and Well-being, Austria introduced a Women’s Health Action Plan in 2017, which highlighted a major lack of information about women’s reproductive health. To date, data on reproductive health knowledge (RHK) and behavior (RHKB), and their relationship with HL remain scarce. The study aimed to examine these relationships among women in Vienna.

**Methods:**

An online cross-sectional survey was conducted in 2023 among women of reproductive age (18–49 years) in Vienna. Collected data included information about sociodemographic characteristics, HL (HLS19-Q12-AT), RHK and RHKB (0–100% scale). Associations between RHK/RHKB and HL, alongside sociodemographic characteristics were examined using linear regression analyses.

**Results:**

Among 386 participating women 41% had limited HL. Mean scores were 71% (SD = 21) for HL, 74% (SD = 16) for RHK, and 78% (SD = 14) for RHKB. The highest rate of incorrect responses concerned egg cell lifespan (61%), human papillomavirus (HPV) consequences (49%) and fertility age (40%). In univariate analyses RHK and RHKB were positively associated with older age and partnership and negatively associated with inadequate HL and certain religious affiliations. The RHKB was further associated with university education and parenthood. Multivariate analyses confirmed age and religion as significant factors.

**Conclusion:**

Our study identified RHK gaps among women in Vienna, showed associations between RHKB and HL and highlighted the importance of information sources. Findings underline the need for targeted interventions to improve reproductive HL.

**Supplementary Information:**

The online version of this article (10.1007/s00508-025-02672-x) contains supplementary material, which is available to authorized users.

## Introduction

Women face a variety of health risks, including biological and genetic predispositions such as hormonal imbalances and menstrual health issues that are specific to women’s health and extend beyond traditional healthcare systems [1–3]. Women often experience delayed diagnoses or misdiagnoses, especially concerning reproductive health, infertility and conditions such as thyroid disorders or endometriosis [1]. In European Union (EU) member states, women are less likely than men to perceive their health status as good or very good (women 65.4%, men 70.5%) [4], despite having a higher life expectancy [5]. Although women live longer and have higher health literacy (HL) than men [6], their healthy life years are similar to that of men (women 62.8 years, men 62.4 years, in 2022) [7].

Low HL is associated in both men and women with poorer health outcomes, greater use of hospital and emergency care and reduced ability to interpret labels and health messages [8, 9]. Among women, low HL was found to negatively impact the understanding of health topics, the ability to navigate the healthcare system, adhere to clinical care plans and achieve positive health outcomes, including reproductive health [10, 11]. Reproductive health encompasses various aspects, such as family planning, contraception, pregnancy and childbirth services, diagnosis and treatment of sexually transmitted infections (STI), safe abortion services, infertility treatment and sexual education [12]. Studies have shown significant links between women’s reproductive health and HL [13, 14], yet this area remains underrepresented in research [2]. Studies in Europe conducted in Hungary [15], Serbia [16] and Poland [17] found that women with higher HL had better reproductive health knowledge (RHK) concerning maternity and menstrual health, STIs and treatment adherence. The HL was also associated with reproductive health behavior (RHB), such as breastfeeding and taking vitamin supplements during pregnancy [11]. Low general HL was associated with poor contraceptive knowledge [18], further underscoring the importance of HL in women’s reproductive health.

Over the past 15 years many member states in the WHO European Region have improved reproductive health indicators, for example, by reducing perinatal and maternal mortality and increasing contraceptive use [2]. International strategies, such as the “Strategy on Women’s Health and Well-being” [2], have been adopted by countries, including Austria, which introduced the national “Action Plan on Women’s Health” in 2017 [19] and has since published relevant reports, including the “Women’s Health Report 2022” [1], Gender Health Report 2024 [20] and “Menstrual Health Report 2024” [21]. These publications have consistently highlighted gaps in data availability, particularly in areas such as menstrual health, sexual and reproductive health [1]; however, to date no studies have systematically examined the relationship between RHK and HL in the Austrian context. Recognizing this gap, the present study examines women’s HL, with a focus on reproductive health.

The Austrian healthcare system is based on a social insurance model, where 99.9% of the population are covered by universal health insurance, with additional private health insurance covering over 30% of the population [22]. Austria reports relatively lower HL levels among surveyed countries, with 56.4% of participants showing limited HL, compared to 31.5% in the Netherlands and an average of 47.6% across 8 participating countries [6]. In 2023 Vienna had 1.98 million residents, of whom 51% were women. Women’s life expectancy in Vienna was 82.7 years [23] of which 58.9 years were in very good or good health, both figures below the Austrian and EU averages [7]. In Vienna, the mean age at first childbirth increased from 27 years in 1991 to 31.7 years in 2022 [23], slightly above the Austrian and EU average of 31.2 years [24]. Considering that health indicators, such as life expectancy, in Vienna fall below the national average and given its diverse cultural and socioeconomic composition compared to other federal states in Austria, Vienna represents a particularly relevant context for studying HL and exploring reproductive HL in greater depth.

The study aimed to examine RHK and RHKB and their association with HL and sociodemographic characteristics among women in Vienna, Austria. Based on the existing literature, the following hypotheses of associations were prespecified: i) women with lower HL have poorer RHK and RHKB compared to women with higher general HL [16, 17], ii) older age is associated with better RHK and RHKB [16, 17], iii) women with children have better RHK and RHKB than women who do not have children [2], iv) women with a higher level of educational attainment have better RHK and RHKB than women with lower levels of education [16, 17], v) women with private supplementary health insurance have better RHK and RHKB than women with only statutory health insurance [1] and vi) women with a migration background have lower RHK and RHKB than women born in Austria [1].

## Methods

The reporting of the study followed the Strengthening the Reporting of Observational Studies in Epidemiology (STROBE) statement for reporting of the study results [25] (Supplementary material Table S1).

### Study population

A cross-sectional survey was conducted on a convenience sample of women aged 18–49 years and living in Vienna. Inclusion criteria were reproductive age (18–49 years), sufficient knowledge of German, access to the online survey and current residence in any district of Vienna. All respondents had to meet all inclusion criteria and provide informed consent to participate in the survey.

### Recruitment

Data collection took place from February 2023 to April 2023 using the SoSci Survey platform. Participants were recruited through advertisements on the authorʼs social media platforms (Facebook, Instagram and LinkedIn), by sending out leaflets and emails to women’s health centers/networks, obstetrics/gynecology (OB/GYN) and general practice clinics, and women’s (sports) associations. Additional social networks were accessed through snowball sampling by contacting the authors’ personal and university networks, including students. The web link and a corresponding QR code for the online questionnaire were included in the advertisements as well as a short summary of the research objective, so that the respondents formed a sample through self-selection.

### Data collection

The questionnaire consisted of four parts with a total of 27 questions: I) sociodemographic characteristics, II) HL questionnaire (HLS19-Q12-AT), III) RHK questionnaire and IV) RHB questionnaire. It was developed using the SoSci survey tool. An additional question asked the study participants about the sources of reproductive health information, with response options referring to employees in the healthcare sector (referred to as health professionals), teachers at schools/universities (referred to as schools/universities), friends, parents, media including digital media (referred to as media) and others. Participation in the survey was voluntary. Participants could not skip any question; however, the answer option “I do not want to answer”, was provided. The questionnaire was pilot tested in November 2022 on a sample of women fulfilling the inclusion criteria. The average duration for participants to complete the questionnaire during the pilot testing was 12 min. Informed consent to participate in the survey was obtained from each study participant prior to the survey. No incentives were offered for completing the survey. The study was approved by the Ethics Committee of the Medical University of Vienna (EK Nr. 2007/2022).

### HLS19-Q12-AT

Following approval of the developers, the German version of the standardized 12-item HLS19-Q12-AT was used to assess women’s general HL [26]. The HLS19-Q12-AT is a psychometrically validated instrument for the assessment of general HL in adults. The questionnaire asks participants to rate the difficulty of 12 given tasks or specific situations related to the healthcare system on a 4-point Likert scale from very easy to very difficult. Scores were calculated according to the official formulae. The total HL score ranges from 0% to 100% and specific categories were defined as excellent (> 83.33%), sufficient (> 66.67% and ≤ 83.33%), problematic (> 50% and ≤ 66.67%) or inadequate (≤ 50%) [6]. The limited HL category combines both problematic and inadequate HL levels.

### Reproductive health knowledge and behavior

The RHK and RHB questions were based on questionnaires used in two previous studies [16, 17]. The authors of the two studies were contacted and asked for permission to translate and adapt the questions to the Austrian context. The RHK section consisted of 11 questions and covered knowledge about women’s menstrual cycle timelines, ovulation, fertility, human papillomavirus (HPV) vaccination and the Papanicolaou (Pap) test. Questions related to behavior asked about regular OB/GYN appointments, participation in Pap screening, interest in reproductive health-related topics and mammography screening. The RHK scores were calculated as the percentage of correct responses to all 11 questions. The RHKB scores were calculated for the 11 RHK questions and the 3 behavior questions as a percentage of correct or desired answers. Desired answers refer to the three questions about behavior. The mammography question was not included in the scoring because only a small percentage of study participants were eligible to answer. The RHK and RHKB scores ranged from 0% to 100%, with higher scores representing better knowledge/behavior.

### Data analysis

Descriptive statistics were used to describe participants’ sociodemographic characteristics, which were coded into the following categorical variables: i) HL (excellent, sufficient, problematic, inadequate), ii) age (18–25, 26–35, 36–49 years), iii) country of birth (Austria, not Austria), iv) the level of education was categorized into university degree and no university degree (combining primary school, apprenticeship/vocational training, vocational secondary school, secondary school equivalent to A‑level examinations “Matura”), v) religion was categorized into no religion, Roman Catholic, other religions (combining Protestant, Old Catholic, Orthodox, Jewish, Muslim) and “I prefer not to answer”, vi) private health insurance (private health insurance, no private health insurance), vii) currently having a partner (yes, no), viii) having children was categorized into yes (combining having one or more children) and no and ix) employment status was categorized into currently working (combining employed, self-employed, marginally employed, maternity leave with a current employment contract), currently not working (combining full-time homemaker, student, unemployed).

Univariate and multivariate linear regressions were conducted to examine the associations between RHK and RHKB and sociodemographic characteristics, HL categories and reproductive health information sources. For the multivariate regression, three separate models for the variables RHK and RHKB were estimated to observe the effect of HL and information source variables on the associations. Model 1 included sociodemographic characteristics, model 2 included sociodemographic characteristics and HL categories and model 3 included information sources. Associations between HL and sociodemographic characteristics (model 1) and information sources (model 4 which did not include the HL categories), were also examined. Multicollinearity was tested using the variance inflation factor (VIF). The individual VIF values varied between 1.10 and 3.99, indicating that multicollinearity was not of concern. No weighting was used to adjust for a nonrepresentative sample. Data analysis was conducted on the complete data. No imputation of missing data was conducted. Data analysis was conducted using Stata version 19.0 (StataCorp LLC, College Station, TX, USA) with a *p*-value of ≤ 0.05 considered statistically significant.

## Results

### Sample characteristics

Out of a total of 534 questionnaire entries, 391 (73.2%) surveys were initiated (provided informed consent), of which 386 participants (72.3%) provided complete responses. The mean age of the participants was 32.1 years (standard deviation, SD: 8.3 years) (Table 1). The majority of the participants (*n* = 303, 78.5%) had a university degree and 21.5% (*n* = 83) had lower educational level (primary school *n* = 2, apprenticeship/vocational training *n* = 11, A‑level examinations equivalent “Matura” *n* = 70). Currently employed or self-employed were 75.1% (*n* = 290) of the respondents, 78.2% were born in Austria (*n* = 302) and 73.6% currently had a partner (*n* = 284). The majority of respondents had no children (*n* = 277, 71.8%), about half reported no religious affiliation (*n* = 177, 45.9%), 38.9% (*n* = 150) reported Roman Catholic religion and 10.6% (*n* = 41) reported other religious associations (Protestant *n* = 25, Orthodox *n* = 3, Islam *n* = 4, other religion *n* = 9).

**Table 1:** Sample characteristics by general health literacy (*n* = 386)

	Sample characteristics		Health literacy	
Variables	*n*	% or mean (SD)	Mean (SD)	*p*-value^1^
*Age (mean, SD)*	386	32.1 years (8.3)	71.1 (21.4)	*–*
*Age categories (years)*				
18–25	98	25.4%	66.7 (20.9)	Ref.
26–35	153	39.6%	68.3 (20.8)	0.558
36–49	135	35.0%	77.3 (21.0)	0.000
*Education*				
No university degree	83	21.5%	64.8 (22.4)	Ref.
University degree	303	78.5%	72.8 (20.8)	0.002
*Employment status*				
Currently employed or self-employed	290	75.1%	73.1 (21.1)	Ref.
Other	96	24.9%	64.7 (21.2)	0.001
*Partnership*				
No partner	99	25.6%	67.1 (21.7)	Ref.
Partner	284	73.6%	72.6 (21.2)	0.029
I do not want to answer*	3	0.8%	55.6 (9.6)	–
*Health insurance*				
No private insurance	238	61.7%	70.1 (21.7)	Ref.
Private insurance	148	38.3%	72.5 (21.2)	0.278
*Children*				
No children	277	71.8%	69.3 (21.2)	Ref.
Children	109	28.2%	75.6 (21.2)	0.008
*Country of birth*				
Outside of Austria	72	18.7%	68.6 (21.6)	Ref.
Austria	302	78.2%	71.5 (21.1)	0.310
I do not want to answer*	12	3.1%	75.1 (26.9)	–
*Religion*				
No religion	177	45.9%	72.4 (20.2)	Ref.
Roman Catholic	150	38.9%	70.5 (22.3)	0.389
Other religion	41	10.6%	69.3 (21.6)	0.420
I do not want to answer*	18	4.7%	66.4 (24.6)	0.265
HL categories*				
Excellent HL	146	37.8%	92.8 (6.6)	–
Sufficient HL	61	21.3%	75.5 (2.6)	–
Problematic HL	97	25.1%	62.3 (4.3)	–
Inadequate HL	82	15.8%	39.4 (9.7)	–
Health information sources*^†^				
Health professionals	271	70.2%	73.1 (21.0)	–
Schools/universities	98	25.4%	70.6 (21.0)	–
Friends	208	53.9%	69.0 (21.3)	–
Parents	97	25%	68.8 (21.2)	–
(Digital) media	303	78.5%	71.2 (21.1)	–
Other	74	19.2%	77.8 (19.7)	–

*ref* reference value, *HL* health literacy, *SD* standard deviation

^1^ Univariate linear regression

* Excluded from the univariate regression

^†^ Multiple answer options possible

On average, participants had a general HL of 71.5% (SD = 21.4; range 16.7–100). Around one third of participants (37.8%) had excellent HL, while 21.3% had sufficient, 25.1% problematic and 15.8% inadequate HL. Limited HL was present in 40.9% of the participants. In univariate analyses, higher HL scores were significantly associated with older age, having a university degree, having a partner and having children (Table 1).

### Reproductive health knowledge and behavior

**Fig. 1:**
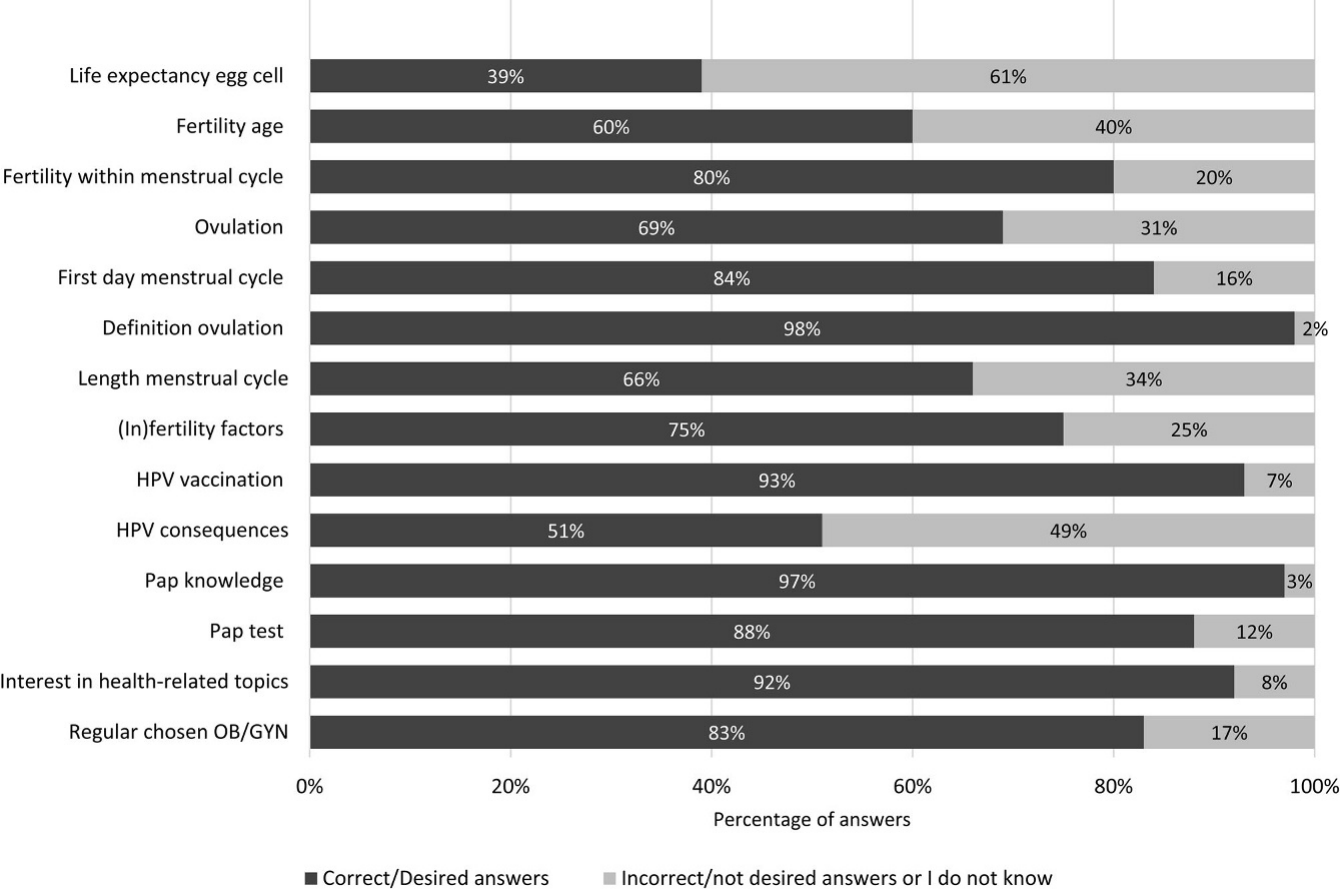
Percentage of correct answers to 11 reproductive health knowledge questions and percentage of desired answers to three reproductive health behavior questions (*n* = 386). Note: *Pap* Papanicolaou test, *OB/GYN* obstetrics/gynecology, *HPV* human papillomavirus

The mean RHK and RHKB scores were 73.9% (SD = 16.5) and 78.2% (SD = 14.2), respectively. Detailed responses revealed substantial knowledge gaps on certain topics regardless of HL level, while other questions were answered correctly by the vast majority (Supplementary material Table S2). As shown in Fig. 1, 61% of the participating women answered incorrectly about egg cell life expectancy, 49% about HPV infection consequences and 40% about the fertile age range. Knowledge gaps were also identified for questions related to fertility, menstrual cycle and ovulation. The question about the definition of ovulation was most frequently correctly answered (98%).

### Univariate analyses

Of the six prespecified hypotheses, univariate analyses confirmed two for both RHK and RHKB (age and HL) and two for RHKB only (having children and education). Older age was significantly associated with higher RHK and RHKB scores. Inadequate HL was significantly associated with lower RHK (−5.93; 95% confidence interval, CI: −10.37, −1.48) and RHKB (−6.27; 95% CI: −10.08, −2.46) scores, compared to excellent HL, whereas problematic HL was not significantly associated. Compared to lower educational attainment, a university degree was positively associated with RHKB (5.43; 95% CI: 2.01, 8.86), but not with RHK. Similarly, having children was statistically significantly associated with higher RHKB, but not with RHK. The two remaining hypotheses, concerning migration background as a binary variable (Austrian versus non-Austrian) and complementary health insurance, were not confirmed in this sample. Additional statistically significant associations included lower RHK and RHKB scores among participants who reported a religious affiliation other than Roman Catholic or no affiliation and higher RHK and RHKB scores among those with a partner (Table 2).

**Table 2:** Univariate associations between reproductive health knowledge and behavior and explanatory variables (*n* = 386)

	Reproductive health knowledge			Reproductive health knowledge and behavior		
Variables	Coefficient (SE)	[95% CI]	*p*-value	Coefficient (SE)	[95% CI]	*p*-value
Age 26–35 years	4.90 (2.11)	[0.74, 9.06]	**0.021**	6.98 (1.79)	[3.46, 10.50]	**<** **0.001**
Age 36–49 years	6.42 (2.17)	[2.15, 10.70]	**0.003**	8.32 (1.83)	[4.71, 11.94]	**<** **0.001**
University degree	3.67 (2.03)	[−0.33, 7.68]	0.073	5.43 (1.74)	[2.01, 8.86]	**0.002**
Current employment	1.75 (1.94)	[−2.06, 5.58]	0.367	4.90 (1.65)	[1.64, 8.16]	**0.003**
Having a partner	4.34 (1.91)	[0.57, 8.12]	**0.024**	6.46 (1.62)	[3.26, 9.67]	**<** **0.001**
Having children	3.07 (1.86)	[−0.58, 6.73]	0.100	3.83 (1.59)	[0.69, 6.97]	**0.017**
Private insurance	0.35 (1.73)	[−3.04, 3.76]	0.836	0.52 (1.49)	[−2.40, 3.45]	0.725
Born in Austria	1.78 (2.17)	[−2.49, 6.05]	0.413	2.87 (1.86)	[−0.78, 6.53]	0.124
Roman Catholic	−0.83 (1.82)	[−4.41, 2.74]	0.648	−0.99 (1.55)	[−4.06, 2.06]	0.522
Other religion	−7.42 (2.84)	[−13.01, −1.83]	**0.009**	−8.35 (2.43)	[−13.14, −3.57]	**0.001**
Sufficient HL	0.67 (2.49)	[−4.23, 5.58]	0.787	0.38 (2.14)	[−3.83, 4.59]	0.859
Problematic HL	−3.05 (2.14)	[−7.27, 1.16]	0.156	−2.46 (1.84)	[−6.07, 1.15]	0.182
Inadequate HL	−5.93 (2.26)	[−10.37, −1.48]	**0.009**	−6.27 (1.93)	[−10.08, −2.46]	**0.001**

*CI* confidence interval, *HL* health literacy, *SE* standard error, *bold values* indicate statistically significant associations (*p* ≤ 0.05)

### Multivariate analyses

Three participants who responded “I do not want to answer” to the question about currently having a partner were excluded from the regression analyses, resulting in a sample size of *n* = 383. Higher RHK and RHKB scores were positively associated with older age and negatively associated with religion other than Roman Catholic, compared to no religious affiliation in all investigated models. Women aged 26–35 years and 36–49 years had more than 7% (7.49; 95% CI: 1.97, 13.01) and 8% (8.35; 95% CI: 1.65, 15.04) higher RHK scores, respectively, compared to women in the youngest age group 18–25 years, when controlling for all other variables (Table 3). Furthermore, being currently employed was negatively associated with RHK (−6.49; 95% CI: −11.76, −1.21, model 3), while having a partner was positively associated with RHKB (3.55; 95% CI: 0.32, 6.79, model 3). Variables related to private health insurance and having children did not show statistically significant associations. Statistically significant associations between RHK or RHKB and HL categories were shown only in model 2, with an approximately 5.5% decrease in the dependent variable score for an inadequate HL relative to an excellent HL. This association was not statistically significant when reproductive health information source was included in the analyses (model 3). In model 3, RHK and RHKB were positively associated with obtaining reproductive health information from health professionals, schools/universities, and other (unspecified) sources; however, obtaining reproductive health information from parents was negatively associated with both RHK and RHKB and HL was significantly associated only with university education and with information sources from health professionals and other (unspecified) sources.

**Table 3:** Associations between reproductive health knowledge and behaviour, health literacy, sociodemographic status and information sources (*n* = 383)

	Reproductive health knowledge	Reproductive health knowledge	Reproductive health knowledge	Reproductive health knowledge and behaviour	Reproductive health knowledge and behaviour	Reproductive health knowledge and behaviour	Health literacy	Health literacy
	Model 1 [95% CI]	Model 2 [95% CI]	Model 3 [95% CI]	Model 1 [95% CI]	Model 2 [95% CI]	Model 3 [95% CI]	Model 1 [95% CI]	Model 4 [95% CI]
*Age (18–35 years—ref.)*								
26–35	6.57^*^ [1.07,12.08]	7.18^*^ [1.69,12.67]	7.49^**^ [1.97,13.01]	6.10^**^ [1.49,10.72]	6.68^**^ [2.09,11.27]	6.99^**^ [2.42,11.55]	−4.74 [−11.81,2.33]	−3.80 [−10.93,3.34]
36–49	8.71^**^ [2.13,15.28]	8.30^*^ [1.75,14.84]	8.35^*^ [1.65,15.04]	7.66^**^ [2.15,13.17]	7.28^**^ [1.81,12.75]	7.28^*^ [1.75,12.81]	4.62 [−3.82,13.06]	5.16 [−3.48,13.80]
*Children (no children—ref.)*	0.13 [−4.83,5.08]	0.33 [−4.59,5.26]	−0.01 [−4.84,4.83]	−0.03 [−4.18,4.12]	0.16 [−3.95,4.28]	−0.21 [−4.21,3.79]	−1.72 [−8.08,4.64]	−1.98 [−8.24,4.27]
*University degree (no degree—ref.)*	1.70 [−2.82,6.23]	0.98 [−3.55,5.50]	1.62 [−2.84,6.08]	2.08 [−1.71,5.87]	1.46 [−2.32,5.24]	2.19 [−1.50,5.88]	6.10^*^ [0.29,11.91]	6.65^*^ [0.93,12.37]
*Currently employed (not currently employed—ref.)*	−5.42^*^ **[−10.76,−0.08]**	−5.90^*^ **[−11.22,−0.58]**	−6.49^*^ **[−11.76,−1.21]**	−2.11 [−6.58,2.37]	−2.56 [−7.01,1.88]	−3.32 [−7.68,1.04]	4.95 [−1.91,11.81]	3.83 [−2.99,10.65]
*Having a partner (no partner—ref.)*	2.58 [−1.43,6.58]	2.22 [−1.77,6.21]	2.09 [−1.82,6.00]	4.06^*^ [0.70,7.41]	3.73^*^ [0.39,7.06]	3.55^*^ [0.32,6.79]	2.72 [−2.42,7.87]	2.38 [−2.67,7.43]
*Religion (no religion—ref.)*								
Roman Catholic	−0.97 [−4.67,2.73]	−0.69 [−4.38,3.00]	0.09 [−3.63,3.81]	−0.69 [−3.80,2.41]	−0.39 [−3.48,2.69]	0.24 [−2.83,3.32]	−1.12 [−5.87,3.63]	−0.80 [−5.60,4.00]
Other religion	−7.37^*^ **[−13.03,−1.71]**	−7.14^*^ **[−12.77,−1.52]**	−7.31^**^ **[−12.85,−1.77]**	−7.79^**^ **[−12.54,−3.05]**	−7.60^**^ **[−12.31,−2.90]**	−7.84^***^ **[12.42,−3.26]**	−2.14 [−9.41,5.12]	−2.99 [−10.16,4.18]
I do not want to answer	3.04 [−4.89,10.96]	3.59 [−4.31,11.50]	2.98 [−4.85,10.81]	2.69 [−3.95,9.32]	3.31 [−3.30,9.92]	2.85 [−3.62,9.33]	−5.00 [−15.17,5.17]	−6.89 [−16.97,3.19]
*Health literacy (excellent—ref.)*								
Sufficient	–	0.57 [−4.29,5.44]	0.99 [−3.81,5.79]	–	0.34 [−3.72,4.41]	0.87 [−3.10,4.84]	–	–
Problematic	–	−2.64 [−6.85,1.57]	−1.89 [−6.07,2.29]	–	−1.68 [−5.20,1.84]	−0.97 [−4.43,2.48]	–	–
Inadequate	–	−5.87^*^ **[−10.45,−1.28]**	−3.87 [−8.49,0.75]	–	−5.57^**^ **[−9.41,-1.73]**	−3.67 [−7.50,0.15]	–	–
*Information sources*†								
Health professionals	–	–	4.48* **[0.83,8.12]**	–	–	5.43*** **[2.41,8.45]**		6.07* **[1.39,10.75]**
Schools/universities	–	–	4.33^*^ **[0.26,8.40]**	–	–	3.72^*^ **[0.35,7.09]**	–	3.26 [−1.99,8.51]
Friends	–	–	0.49 [−2.95,3.92]	–	–	0.25 [−2.59,3.09]	–	−3.45 [−7.87,0.96]
Parents	–	–	−4.75^*^ **[−8.79,-0.72]**	–	–	−4.74^**^ **[−8.08,−1.40]**	–	0.86 [−4.35,6.08]
Media	–	–	2.95 [−1.17,7.06]	–	–	2.89 [−0.52,6.29]	–	2.87 [−2.44,8.18]
Other	–	–	5.12^*^ [0.85,9.40]	–	–	3.56^*^ [0.03,7.10]	–	8.37^**^ [2.90,13.84]
*Constant*	69.88^***^ [64.85,74.91]	72.57^***^ [66.92,78.23]	64.88^***^ [57.03,72.73]	70.78^***^ [66.56,75.00]	73.09^***^ [68.36,77.82]	65.57^***^ [59.07,72.06]	61.77^***^ [55.30,68.23]	54.89^***^ [45.60,64.18]
*Observations*	383	383	383	383	383	383	383	383
*R*^*2*^	0.06	0.05	0.08	0.11	0.10	0.20	0.05	0.12

*ref* reference category

† Reference category refers to not selecting the respective category as an information source

Bold values indicate statistically significant associations, * *p* ≤ 0.05, ** *p* ≤ 0.01, *** *p* ≤ 0.001; numbers in square brackets represent 95% confidence intervals

## Discussion

This study aimed to investigate women’s reproductive health knowledge in Vienna and its relationship with HL and socioeconomic characteristics. It is among the few international studies exploring HL in relation to RHK and RHKB and the first to be conducted in Austria. Despite a highly educated study sample (78% with a university degree compared to 21.9% nationally [27]), 41% of the participants demonstrated limited HL, consistent with HLS Consortium survey findings [6].

The results showed a statistically significant association between inadequate HL and lower RHK and RHB, suggesting that women with higher HL also have better knowledge and behavior concerning reproductive health; however, the association with HL became weaker when information sources on reproductive health topics were included in regression models, suggesting that the source of health information may play a more critical role in the investigated sample. Obtaining information from health professionals and schools/universities was positively associated with RHK, while information obtained from parents was negatively associated. Consistent with previous studies [15–17] better RHK and RHB were associated with older age. Religious affiliation also emerged as a significant factor related to RHK and RHKB. Having a partner was associated with RHKB, which may be related to family planning. Age did not show a statistically significant association with HL, while university education emerged as the strongest predictor of HL.

Consistent with other studies [11, 16–18] participants in the present study demonstrated fragmented knowledge and behavior related to women’s reproductive health, highlighting the need for targeted public health interventions. Previous studies have found HL to be associated with knowledge of the exact fertile period within the menstrual cycle [16, 18, 28]. In the present study 60.6% of the participants did not know the lifespan of an egg cell and only slightly more women with higher HL answered this question correctly compared to those women with lower HL. Additionally, one in five women could not identify the most fertile phase of the cycle and nearly half did not know the general fertile period in a woman’s life. These gaps show areas for improvement in reproductive HL in Vienna. Similar knowledge gaps related to fertile phase, menstruation and ovulation were identified in a study of gynecological outpatients aged 15–50 years in Austria [28].

The findings of this study have important policy implications. Limited knowledge on certain topics, such as ovulation, indicates gaps in reproductive health education and information. Targeted culturally sensitive educational programs tailored for all age groups and health professionals are needed [29]. Although our study population may include groups with similar cultural backgrounds (e.g., German-speaking or Christian participants), religion still emerged as a significant factor associated with reproductive health knowledge and behavior. Introducing evidence-based reproductive health education early in formal schooling could help reduce the stigma around topics such as infertility, gender-based violence and menstrual health [30]. This study also emphasizes the critical role of information sources about reproductive health. Access to high-quality and appropriate health information, tailored to different HL levels, is important for improving reproductive health outcomes.

Although the present study provides valuable insights into the relationship between RHK, RHKB, and HL among women in Vienna, its limitations should be acknowledged. Self-reporting can introduce response bias. Responses might have been influenced by socially desirable patterns, which could affect the reliability of the findings. To a large extent, the questionnaire was developed specifically for this study, limiting the comparability with existing research; however, HL was assessed using a standardized instrument which enables international comparisons. The cross-sectional study design does not allow for causal inference. While associations between HL, RHK, and RHKB were identified, it is not possible to determine causality or the direction of these relationships. Furthermore, the convenience sampling method resulted in a study sample that is not fully representative of the female population in Vienna. Some of the population groups, such as women with lower educational status, diverse migration backgrounds and religious affiliations, as well as those without access to the internet, were underrepresented, limiting the generalizability of the findings. Additionally, although more detailed answer options were available for variables such as education, employment and religion, the small number of responses in some categories required grouping them into broader subcategories, which restricted the ability to examine their associations with RHKB in more detail. The migration background variable included two response options limited to “born in Austria” and “born outside of Austria”, which limited the scope of the analysis. Future research should explore these associations among more diverse groups, particularly in relation to religion and migration background. Moreover, while the study highlights the importance of information sources on reproductive health, it did not comprehensively assess their quality and accessibility, which are likely crucial for reproductive health literacy and should be addressed in future studies. Potentially relevant information about the study participants, such as desire to have children, decision to remain childless, or health status, was not part of the survey. While this study provides a foundation for future research, larger, longitudinal and representative studies are needed to further investigate HL, RHK, their relationship and the effectiveness of interventions designed to improve them.

In conclusion, the study provides insights into the relationship between HL and RHKB among women in Vienna, within the context of key demographic and socioeconomic characteristics. The findings underscore the complexity of these relationships and identify potentially relevant variables, such as religion and information sources, that warrant deeper examination in future research.

## Supplementary Information

STROBE checklist; Responses to questions by health literacy; Questionnaire

## Data Availability

The questionnaire used in the study and the dataset are available on request.
