# Supplementary material for: Association between health literacy and reproductive health knowledge and behavior: A cross-sectional survey among women of reproductive age in Vienna
Source: Wien Klin Wochenschr. 2026 Jan 3;138(11-12):347–56. doi: 10.1007/s00508-025-02672-x (PMC13279678; doi:10.1007/s00508-025-02672-x)
Supplement: Supplementary file 1 — STROBE checklist; Responses to questions by health literacy; Questionnaire [file 508_2025_2672_MOESM1_ESM.docx]

# **Supplementary material**

Table S1. STROBE Statement—Checklist of items that should be included in reports of ***cross-sectional studies***

|  | Item No | Recommendation |  |
| --- | --- | --- | --- |
| **Title and abstract** | 1 | (*a*) Indicate the study’s design with a commonly used term in the title or the abstract | √ |
|  |  | (*b*) Provide in the abstract an informative and balanced summary of what was done and what was found | √ |
| Introduction | | |  |
| Background/rationale | 2 | Explain the scientific background and rationale for the investigation being reported | √ |
| Objectives | 3 | State specific objectives, including any prespecified hypotheses | √ |
| Methods | | |  |
| Study design | 4 | Present key elements of study design early in the paper | √ |
| Setting | 5 | Describe the setting, locations, and relevant dates, including periods of recruitment, exposure, follow-up, and data collection | √ |
| Participants | 6 | (*a*) Give the eligibility criteria, and the sources and methods of selection of participants | √ |
| Variables | 7 | Clearly define all outcomes, exposures, predictors, potential confounders, and effect modifiers. Give diagnostic criteria, if applicable | √ |
| Data sources/ measurement | 8* | For each variable of interest, give sources of data and details of methods of assessment (measurement). Describe comparability of assessment methods if there is more than one group | √ |
| Bias | 9 | Describe any efforts to address potential sources of bias | - |
| Study size | 10 | Explain how the study size was arrived at | √ |
| Quantitative variables | 11 | Explain how quantitative variables were handled in the analyses. If applicable, describe which groupings were chosen and why | √ |
| Statistical methods | 12 | (*a*) Describe all statistical methods, including those used to control for confounding | √ |
|  |  | (*b*) Describe any methods used to examine subgroups and interactions | √ |
|  |  | (*c*) Explain how missing data were addressed | √ |
|  |  | (*d*) If applicable, describe analytical methods taking account of sampling strategy | Not applicable |
|  |  | (*e*) Describe any sensitivity analyses | Not applicable |
| Results | | |  |
| Participants | 13* | (a) Report numbers of individuals at each stage of study—eg numbers potentially eligible, examined for eligibility, confirmed eligible, included in the study, completing follow-up, and analysed | √ |
|  |  | (b) Give reasons for non-participation at each stage | Not applicable |
|  |  | (c) Consider use of a flow diagram | - |
| Descriptive data | 14* | (a) Give characteristics of study participants (eg demographic, clinical, social) and information on exposures and potential confounders | √ |
|  |  | (b) Indicate number of participants with missing data for each variable of interest | √ |
| Outcome data | 15* | Report numbers of outcome events or summary measures | √ |
| Main results | 16 | (*a*) Give unadjusted estimates and, if applicable, confounder-adjusted estimates and their precision (eg, 95% confidence interval). Make clear which confounders were adjusted for and why they were included | √ |
|  |  | (*b*) Report category boundaries when continuous variables were categorized | √ |
|  |  | (*c*) If relevant, consider translating estimates of relative risk into absolute risk for a meaningful time period | Not applicable |
| Other analyses | 17 | Report other analyses done—eg analyses of subgroups and interactions, and sensitivity analyses | Not applicable |
| Discussion | | |  |
| Key results | 18 | Summarise key results with reference to study objectives | √ |
| Limitations | 19 | Discuss limitations of the study, taking into account sources of potential bias or imprecision. Discuss both direction and magnitude of any potential bias | √ |
| Interpretation | 20 | Give a cautious overall interpretation of results considering objectives, limitations, multiplicity of analyses, results from similar studies, and other relevant evidence | √ |
| Generalisability | 21 | Discuss the generalisability (external validity) of the study results | √ |
| Other information | | |  |
| Funding | 22 | Give the source of funding and the role of the funders for the present study and, if applicable, for the original study on which the present article is based | Not appliable |

*Give information separately for exposed and unexposed groups.

Table S2. Responses to reproductive health knowledge and reproductive health behaviour questions, grouped by health literacy categories (n=386)

| **Variables** | **N (%)** | **Answer categories**  **(correct or desired answers in bold)** | **Percentage of correct answers by HL categories** |
| --- | --- | --- | --- |
| **Reproductive health knowledge** | | | |
| **Pap knowledge** *“Do you know what a pap smear is and where you can have it done?”* | | | |
|  | 374 (96.9%) | **Correct answer** | Excellent 97.3% Sufficient 96.3% Problematic 97.9% Inadequate 95.1% |
|  | 12 (3.1%) | Incorrect answer/ I do not know |  |
| **HPV consequences (multiple selection)** *“Do you know what consequences an infection with human papilloviruses (HPV) can have?”* | | | |
|  | 197 (51.0%) | **Correct answer** | Excellent 56.2% Sufficient 62.3% Problematic 44.3% Inadequate 41.5% |
|  | 189 (49.0%) | Incorrect answer/ I do not know |  |
| **HPV vaccination** *“Is there a vaccination against HPV?”* | | | |
|  | 361 (93.5%) | **Correct answer** | Excellent 97.2% Sufficient 95.1% Problematic 89.7% Inadequate 90.2% |
|  | 25 (6.5%) | Incorrect answer/ I do not know |  |
| **(In)fertility factors** *“Do factors such as too many vegetables/fruits per day, non-professional sporting activities and regular sexual intercourse have a detrimental effect on human fertility?”* | | | |
|  | 288 (74.6%) | **Correct answer** | Excellent 78.1% Sufficient 80.3% Problematic 77.3% Inadequate 61.0% |
|  | 98 (25.4%) | Incorrect answer/ I do not know |  |
| **Length menstrual cycle** *“How long does a normal menstrual cycle last?”* | | |  |
|  | 255 (66.1%) | **Correct answer** | Excellent 69.2% Sufficient 67.2% Problematic 62.3% Inadequate 63.4% |
|  | 131 (33.9%) | Incorrect answer/ I do not know |  |
| **Definition ovulation** *“What describes ovulation?”* | | |  |
|  | 379 (98.2%) | **Correct answer** | Excellent 98.6% Sufficient 96.7% Problematic 96.9% Inadequate 100% |
|  | 7 (1.8%) | Incorrect answer/ I do not know |  |
| **First day menstrual cycle** *“Which day counts as day 1 of your menstrual cycle?”* | | | |
|  | 324 (83.9%) | **Correct answer** | Excellent 87.7% Sufficient 88.5% Problematic 80.4% Inadequate 78.1% |
|  | 62 (16.1%) | Incorrect answer/ I do not know |  |
| **Ovulation** *“At what point in the cycle does ovulation occur?”* | | | |
|  | 267 (69.2%) | **Correct answer** | Excellent 68.5% Sufficient 63.9% Problematic 73.2% Inadequate 69.5% |
|  | 119 (30.8%) | Incorrect answer/ I do not know |  |
| **Fertility within menstrual cycle** *“Which phase of the menstrual cycle is the most fertile?”* | | | |
|  | 308 (79.8%) | **Correct answer** | Excellent 80.8% Sufficient 86.9% Problematic 76.3% Inadequate 76.8% |
|  | 78 (20.2%) | Incorrect answer/ I do not know |  |
| **Fertility age** *“In what period of their lives are women theoretically fertile?”* | | | |
|  | 232 (60.1%) | **Correct answer** | Excellent 61.0% Sufficient 63.9% Problematic 62.9% Inadequate 52.4% |
|  | 154 (39.9%) | Incorrect answer/ I do not know |  |
| **Life expectancy egg cell** *“How long does a healthy egg live under favorable conditions?”* | | | |
|  | 151 (39.1%) | **Correct answer** | Excellent 39.0% Sufficient 41.0% Problematic 38.1% Inadequate 39.0% |
|  | 239 (60.9%) | Incorrect answer/ I do not know |  |
| **Reproductive health behavior** | | | |
| **Chosen OB/GYN** *“Do you have a gynecologist you trust and to whose practice you go regularly?”* | | | |
|  | 320 (82.9%) | **Yes** | Excellent 87.0% Sufficient 86.9% Problematic 83.5% Inadequate 71.5% |
|  | 66 (17.1%) | No/ I do not want to provide an answer |  |
| **Interest in health-related topics** *“Do you follow health-related topics?”* | | | |
|  | 355 (92.0%) | **Yes** | Excellent 93.2% Sufficient 91.8% Problematic 95.9% Inadequate 85.4% |
|  | 31 (8.0%) | No |  |
| **Pap test** *“Have you already had a PAP smear test carried out?”* | | | |
|  | 341 (88.3%) | **Yes** | Excellent 93.1% Sufficient 91.8% Problematic 85.6% Inadequate 80.5% |
|  | 45 (11.7%) | No/I do not know |  |

Note: Correct or desirable answers marked bold; HL – health literacy

**QUESTIONNAIRE**

**Liebe Studienteilnehmerin, im folgenden Teil der Umfrage werden sozio-demografisch-ökonomische Merkmale abgefragt. Alle Angaben werden voll-anonymisiert verarbeitet und sind nicht auf Sie zurückzuführen.**

**Sozio-demografisch-ökonomische Fragen:**

1. Wie alt sind Sie?

Mögliche Antworten: 18-49

1. In welchem Bezirk wohnen Sie?

Mögliche Antworten: 1-23

1. In welchem Land wurden Sie geboren?
2. Österreich
3. In einem anderen Land?
4. Ich möchte keine Angabe machen
5. Was ist Ihr höchster Bildungsabschluss?
6. Pflichtschulabschluss
7. Lehre / BMS
8. Matura
9. Hochschule / Universität
10. Welcher der folgenden Gruppen würden Sie sich vorwiegend zuordnen?
11. Unselbständig erwerbstätig (Arbeiterin, Angestellte, Vertragsbedienstete, Beamtin)
12. Selbständig erwerbstätig
13. Geringfügig erwerbstätig
14. Mutterschutz/ Elternkarenz (aufrechtes Dienstverhältnis)
15. Ausschließlich haushaltsführend (Hausfrau)
16. Arbeitslos
17. Schülerin, Studentin
18. Andere
19. Wie würden Sie ihr Religionsbekenntnis beschreiben?
20. Römisch-katholisch
21. Evangelisch
22. Altkatholisch
23. Orthodox
24. Israelitisch
25. Islamisch
26. Andere Religion
27. Keine Religion
28. Ich möchte keine Angabe machen.
29. Haben Sie eine private Krankenversicherung als freiwillige Zusatzversicherung abgeschlossen?
30. Ja
31. Nein
32. Leben Sie in einer Partnerschaft?
33. Ja
34. Nein
35. Ich möchte keine Angabe machen.
36. Wie viele Kinder haben Sie?
37. Keine Kinder
38. 1-2 Kinder
39. Mehr als 2 Kinder
40. Ich möchte keine Angabe machen

**Reproduktive Gesundheit (Verhalten und Verständnis)**

**Liebe Studienteilnehmerin, im Folgenden beschäftigt sich die Umfrage mit reproduktivem Gesundheitsverhalten. Es geht um Ihre persönlichen Verhaltensweisen in bestimmten Situationen.
Alle Daten werden voll-anonymisiert ausgewertet und sind nicht auf Sie zurückzuführen.**

**Reproduktives Gesundheitsverhalten**

1. Haben Sie eine Gynäkologin/einen Gynäkologen Ihres Vertrauens, zu dessen Ordination Sie regelmäßig gehen?
2. Ja
3. Nein
4. Ich möchte keine Angabe machen.
5. Verfolgen Sie gesundheitsbezogene Themen?
6. Ja
7. Nein
8. Was sind Ihre Informationsquellen rund um das Thema Ihrer (reproduktiven) Gesundheit? [Hinweis: Sie können hier mehrere Antworten geben]
9. Mitarbeitende im Gesundheitswesen
10. Unterrichtseinheiten in der Schule/Uni
11. Freund*innen
12. Eltern
13. (digitale) Medien
14. Andere
15. Verwenden Sie Verhütungsmittel?
16. Ja
17. Nein
18. Ich möchte keine Angabe machen.
19. Sind Sie schon einmal zur Mammographie gegangen?
20. Ja
21. Nein
22. Nichtzutreffend, weil ich unter 45 Jahre alt bin
23. Ich möchte keine Angabe machen.

14.1 Waren Sie dort…

1. freiwillig
2. im Rahmen eines organisierten Screenings ab dem 45. Lebensjahr (schriftliche Einladung)
3. auf ärztliche Anweisung
4. Ich weiß es nicht.
5. Ich möchte keine Angabe machen.
6. Haben Sie den PAP-Abstrich bereits einmal vornehmen lassen?
7. Ja
8. Nein
9. Ich weiß es nicht.
10. Ich möchte keine Angabe machen.

**Liebe Studienteilnehmerin, im letzten Umfrageteil geht es um das Wissen zu reproduktiver Gesundheit. Es geht nicht um Ihre persönlichen Erfahrungen, sondern rein um Ihr Wissen zu den einzelnen Fragen. Alle Daten werden voll-anonymisiert ausgewertet und sind nicht auf Sie zurückzuführen.**

**Reproduktives Gesundheitsverständnis**

1. Wissen Sie, was der PAP-Abstrich ist und wo Sie ihn vornehmen lassen können?
2. Gynäkologischer Eingriff: Schwangerschaftsabbruch
3. Gynäkologischer Routinetest: Zellabstrich vom Gebärmutterhals zur Krebsvorsorge
4. Hausärztlicher Diagnostiktest für bösartige Krebszellen in der Blase
5. Ich weiß es nicht.
6. Wissen Sie, welche Folgen eine Infektion mit den humanen Papillomviren (HPV) haben kann? [Hinweis: Sie können hier mehrere Antworten geben]
7. Erhöhtes Krebsrisiko
8. Entzündungen und Hautveränderungen (z.B. Genitalwarzen)
9. Haarausfall
10. Ich weiß es nicht
11. Gibt es eine Impfung gegen HPV?
12. Ja
13. Nein
14. Ich weiß es nicht.
15. Wirken sich Faktoren wie zu viel Gemüse/Obst pro Tag, nicht-professionelle sportliche Aktivitäten, regelmäßiger Geschlechtsverkehr nachteilig auf die menschliche Fruchtbarkeit aus?
16. Ja
17. Nein
18. Ich weiß es nicht.
19. Wie lang dauert ein normaler Menstruationszyklus?
20. Exakt 28 Tage
21. 21-35 Tage
22. 26-28 Tage
23. Keine der oben genannten Antworten ist korrekt
24. Ich weiß es nicht
25. Was beschreibt die Ovulation?
26. Den Eisprung
27. Ein anderer Begriff für Menstruationszyklus
28. Die Periode
29. Ich weiß es nicht
30. Welcher Tag zählt als Tag 1 Ihres Menstruationszyklus?
31. Erster Tag der Periode
32. Tag der Ovulation (Eisprung)
33. Letzter Tag der Periode
34. Keine der oben genannten Antworten ist korrekt
35. Ich weiß es nicht
36. Zu welchem Zeitpunkt im Zyklus geschieht die Ovulation (der Eisprung)?
37. Ca. 14 Tage vor der Periode
38. Genau am 14. Tag des Menstruationszyklus,
39. Exakt in der Mitte des Zyklus – unabhängig von der Länge,
40. Ich weiß es nicht
41. Welche Phase des Menstruationszyklus ist die fruchtbarste?
42. Lutealphase
43. Follikuläre Phase
44. Ovulationsphase
45. Menstruationsphase
46. Ich weiß es nicht.
47. In welchem Zeitraum ihres Lebens sind Frauen theoretisch fruchtbar?
48. Im Alter zwischen 15-35 Jahren
49. Von der ersten Menstruation bis zur letzten
50. Vom ersten Eisprung bis zum letzten (richtig),
51. Ich weiß es nicht.
52. Wie lange lebt eine gesunde Eizelle unter günstigen Bedingungen?
53. Vom Eisprung bis zum Zyklusende
54. Vom ersten Tag der Periode bis zum 14. Tag des Zyklus
55. 12-24 Stunden
56. 96 Stunden
57. Ich weiß es nicht.
